# Corrigendum: TALPID3/KIAA0586 Regulates Multiple Aspects of Neuromuscular Patterning During Gastrointestinal Development in Animal Models and Human

**DOI:** 10.3389/fnmol.2022.871557

**Published:** 2022-04-29

**Authors:** Jean Marie Delalande, Nandor Nagy, Conor J. McCann, Dipa Natarajan, Julie E. Cooper, Gabriela Carreno, David Dora, Alison Campbell, Nicole Laurent, Polychronis Kemos, Sophie Thomas, Caroline Alby, Tania Attié-Bitach, Stanislas Lyonnet, Malcolm P. Logan, Allan M. Goldstein, Megan G. Davey, Robert M. W. Hofstra, Nikhil Thapar, Alan J. Burns

**Affiliations:** ^1^Centre for Immunobiology, Barts and The London School of Medicine and Dentistry, Queen Mary University of London, London, United Kingdom; ^2^Stem Cells and Regenerative Medicine, Birth Defects Research Centre, UCL Great Ormond Street Institute of Child Health, London, United Kingdom; ^3^Department of Anatomy, Histology and Embryology, Semmelweis University, Budapest, Hungary; ^4^Developmental Biology and Cancer Program, Birth Defects Research Centre, UCL Great Ormond Street Institute of Child Health, London, United Kingdom; ^5^Department of Paediatric Surgery, Christchurch Hospital, Christchurch, New Zealand; ^6^Génétique et Anomalies du Développement, Université De Bourgogne, Service d'Anatomie Pathologique, Dijon, France; ^7^Laboratory of Embryology and Genetics of Congenital Malformations, INSERM UMR 1163 Institut Imagine, Paris, France; ^8^Department of Genetics, Hôpital Necker-Enfants Malades, Assistance Publique Hôpitaux de Paris (AP-HP), Paris, France; ^9^Paris Descartes, Sorbonne Paris Cité, Paris, France; ^10^Randall Division of Cell and Molecular Biophysics, King's College London, London, United Kingdom; ^11^Department of Pediatric Surgery, Massachusetts General Hospital, Harvard Medical School, Boston, MA, United States; ^12^Division of Developmental Biology, The Roslin Institute, The University of Edinburgh, Edinburgh, United Kingdom; ^13^Department of Clinical Genetics, Erasmus University Medical Center, Rotterdam, Netherlands; ^14^Division of Neurogastroenterology and Motility, Department of Gastroenterology, Great Ormond Street Hospital for Children NHS Foundation Trust, London, United Kingdom; ^15^Gastrointestinal Drug Discovery Unit, Takeda Pharmaceuticals International, Inc., Cambridge, MA, United States

**Keywords:** TALPID3, KIAA0586, Sonic Hedgehog, enteric nervous system, neural crest cell, gastrointestinal tract, short-rib polydactyly syndrome, Joubert syndrome

In the original article, there was an error. Figures 2, 3, 4 and 5 were referenced incorrectly. All references to Figure 5 should have been Figure 2, all references to Figure 4 should have been Figure 3, all references to Figure 3 should have been Figure 4, all references to figure 2 should have been Figure 5. The authors apologize for this error and state that this does not change the scientific conclusions of the article in any way. The original article has been updated.

## Publisher's Note

All claims expressed in this article are solely those of the authors and do not necessarily represent those of their affiliated organizations, or those of the publisher, the editors and the reviewers. Any product that may be evaluated in this article, or claim that may be made by its manufacturer, is not guaranteed or endorsed by the publisher.

