# Supplementary figures and images for: TALPID3/KIAA0586 Regulates Multiple Aspects of Neuromuscular Patterning During Gastrointestinal Development in Animal Models and Human
Source: Front Mol Neurosci. 2021 Dec 23;14:757646. doi: 10.3389/fnmol.2021.757646 (PMC8733242; doi:10.3389/fnmol.2021.757646)

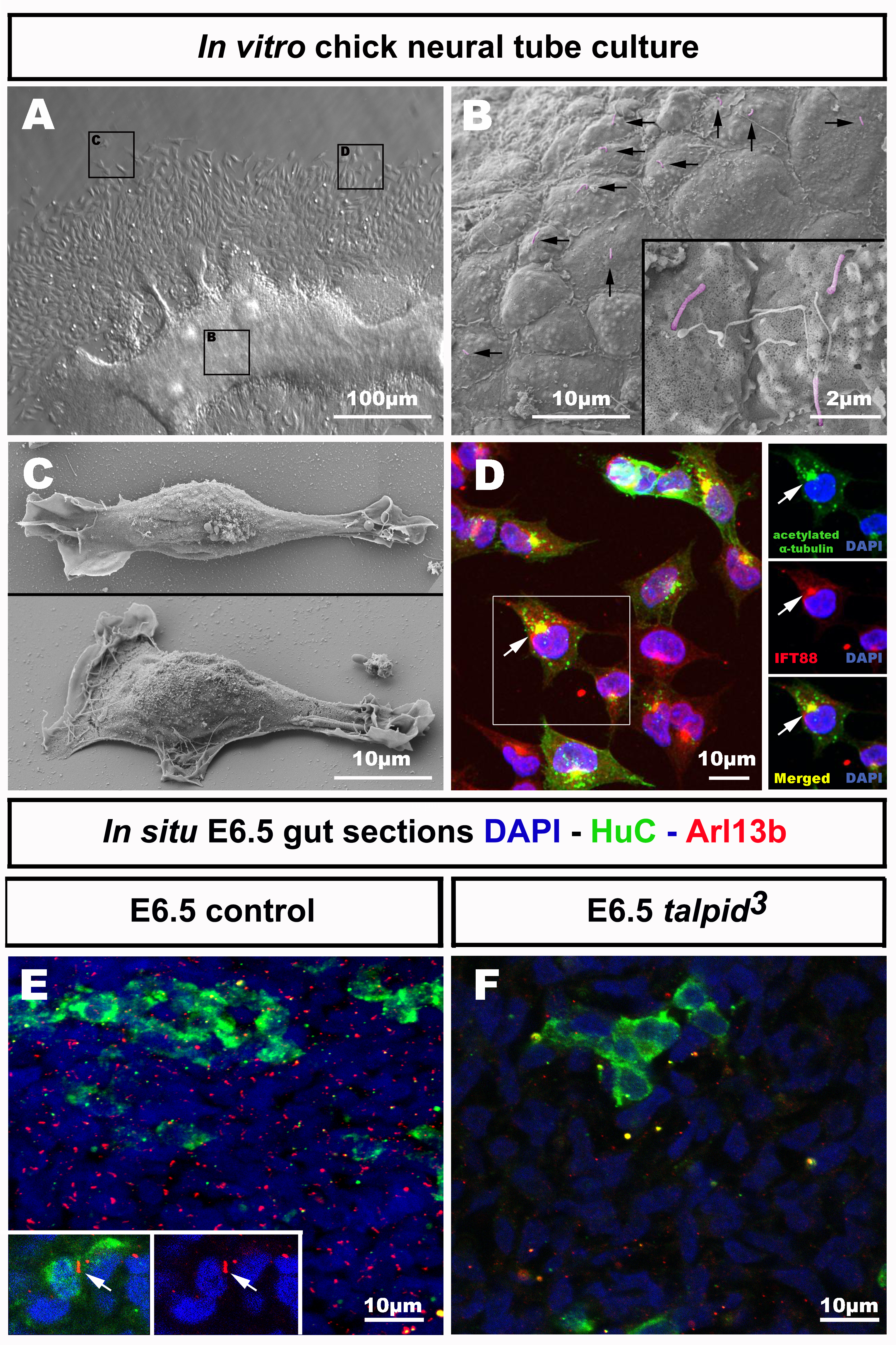

Supplement: Supplementary Figure 1 — Migrating ENCCs do not extend a primary cilium. (A–C) Scanning electron microscopy of neural tube culture and migrating vagal neural crest after 18 h culture. (A) Low magnification of picture shows neural tube and migrating vagal neural crest spreading out. (B) Close up of the neural tube shows primary cilia on cells (pseudocolored in purple, black arrows). (C) Close up of migrating vagal NCC shows no primary cilium. (D) Immunofluorescent staining for acetylated α-tubulin (green) and intraflagellar transport protein 88 (red) shows colocalization at the centrosome and no primary cilium on migrating vagal NCC (white arrow). (E) In vivo staining for the primary cilia with Arl13b on E6.5 gut sections shows signal in all mesenchymal gut cells. In migrating HuC+ (green) ENCCs, 11% show an extended primary cilium (inset; 10 out of 85 HuC+ cells counted, n = 7 sections). (F) No primary cilia staining is observed in any cell type on talpid3 E6.5 gut section. [file Image_1.jpeg]

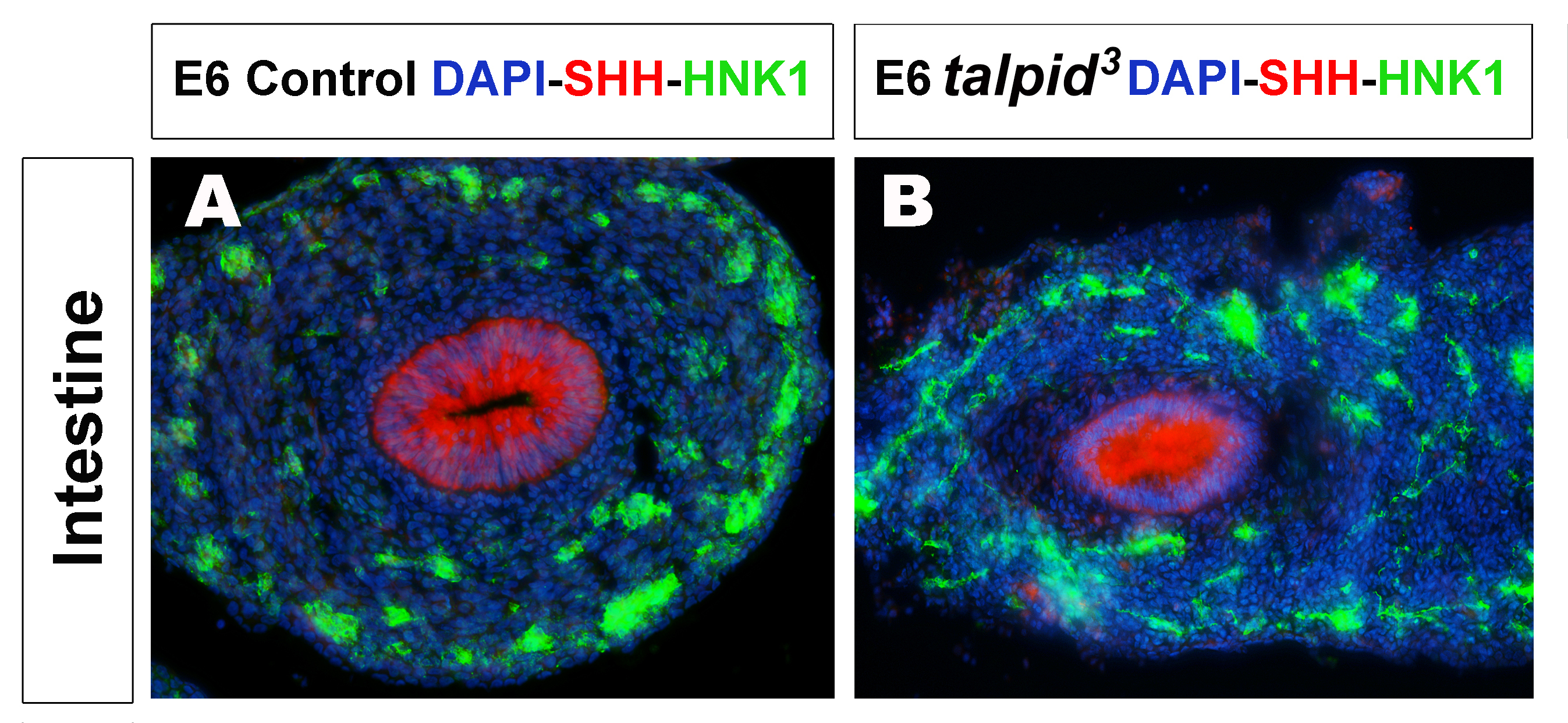

Supplement: Supplementary Figure 2 — The protein Sonic Hedgehog is expressed in both control and talpid3 intestine. (A,B) Immunofluorescent staining of intestine with SHH and HNK1 in (A) E6.5 control and (B) talpid3 mutant embryo. SHH (red) is expressed in the epithelium of both samples. Scattered HNK1 + ENCC (green) are observed in the intestine of talpid3 mutant embryo. [file Image_2.jpeg]

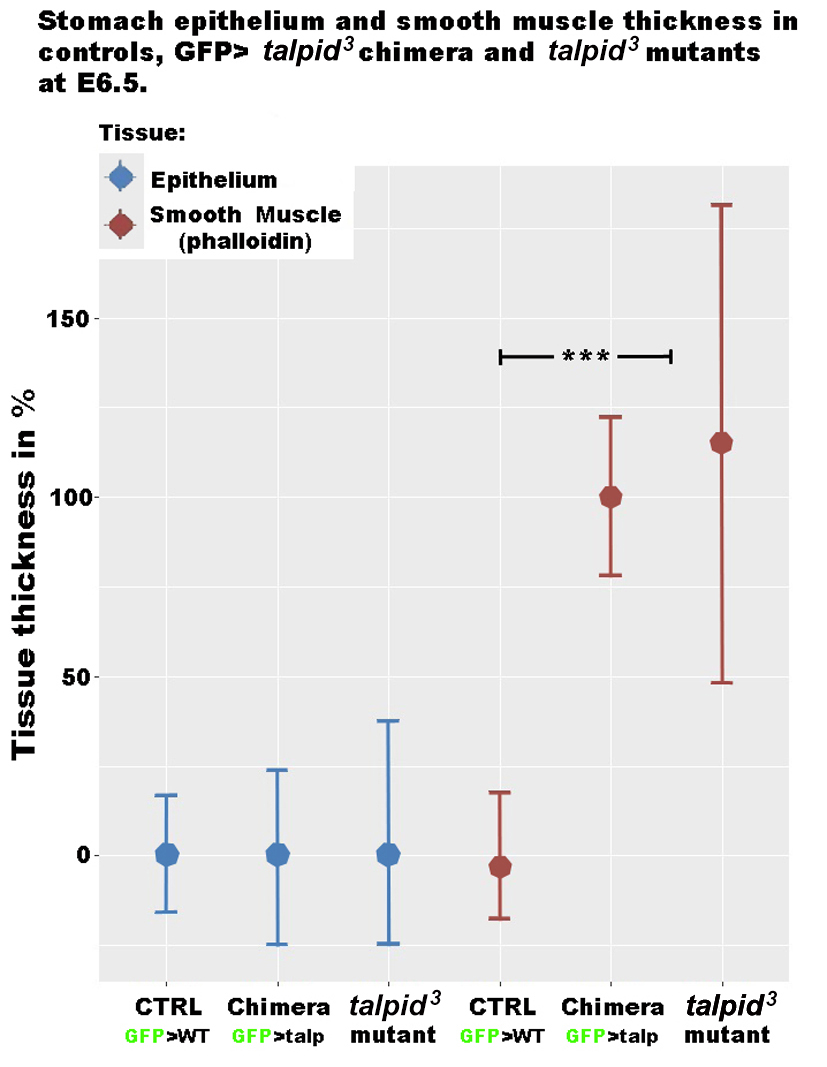

Supplement: Supplementary Figure 3 — Transplantation of wild type ENCCs does not rescue the muscle phenotype in GFP > talpid3 chimeric E6.5 embryo. Measurements of epithelium (DAPI) and smooth muscle (Phalloidin) thickness in stomach and intestine sections were normalized to baseline. At E6.5 there was no statistical difference between the thickness of the epithelium of controls versus GFP > talpid3 chimera or talpid3 mutants. Thickness of the smooth muscle, as measured by phalloidin was increased 100% in the chimera and 115% in the mutant compared to GFP > wild type control transplant. Measurements of phalloidin thickness in GFP > talpid3 chimera and mutant were statistically equivalent and they were both statistically significant from the baseline control (***p < 0.001). ctrl, control. [file Image_3.jpeg]

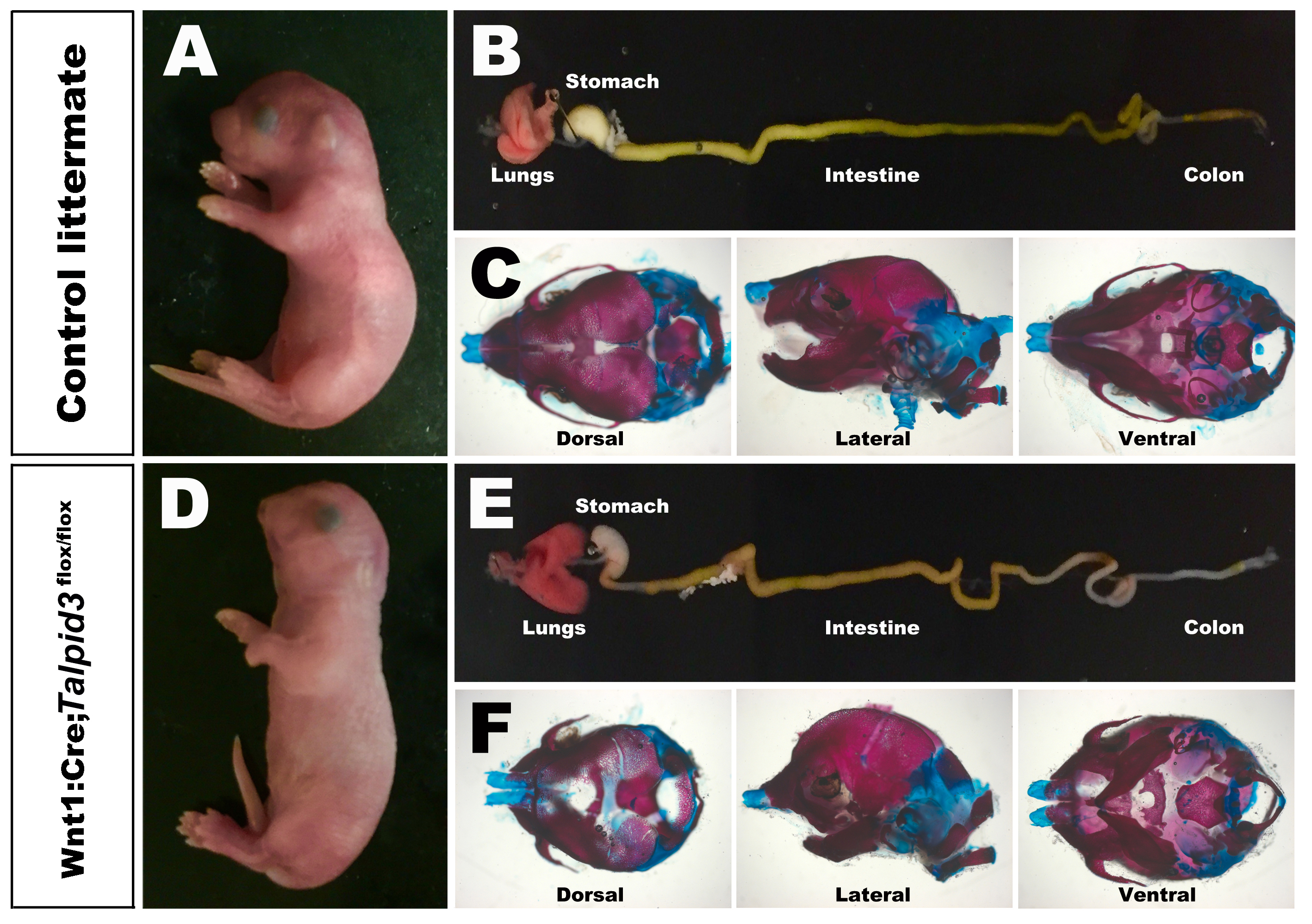

Supplement: Supplementary Figure 4 — Cranial and GI tract phenotypes of Wnt1:Cre;Talpid3fl/fl P0 mice and control littermate. (A,D) Gross phenotype of (A) control littermate and (D) Wnt1:Cre;Talpid3fl/fl P0 pups. (B,E) Gross morphology of the dissected gastrointestinal tract and lungs of (B) control littermate and (E) Wnt1:Cre;Talpid3fl/fl P0 pups. Mutant littermates show grossly normal GI tract, red colored uninflated lungs as well as craniofacial abnormalities such as cleft pallet, short snot and brachycephaly. (C,F) Skeletal staining of the skulls of (C) control littermate and (F) Wnt1:Cre;Talpid3fl/fl P0 pups. Red indicates bone and blue indicates cartilage. (F) Wnt1:Cre;Talpid3fl/fl skull shows frontonasal hypoplasia, hypoplastic NCC derivatives, micrognathia, facial cleft-partitioning of the nasal cartilage and underdeveloped sagittal suture. [file Image_4.jpeg]

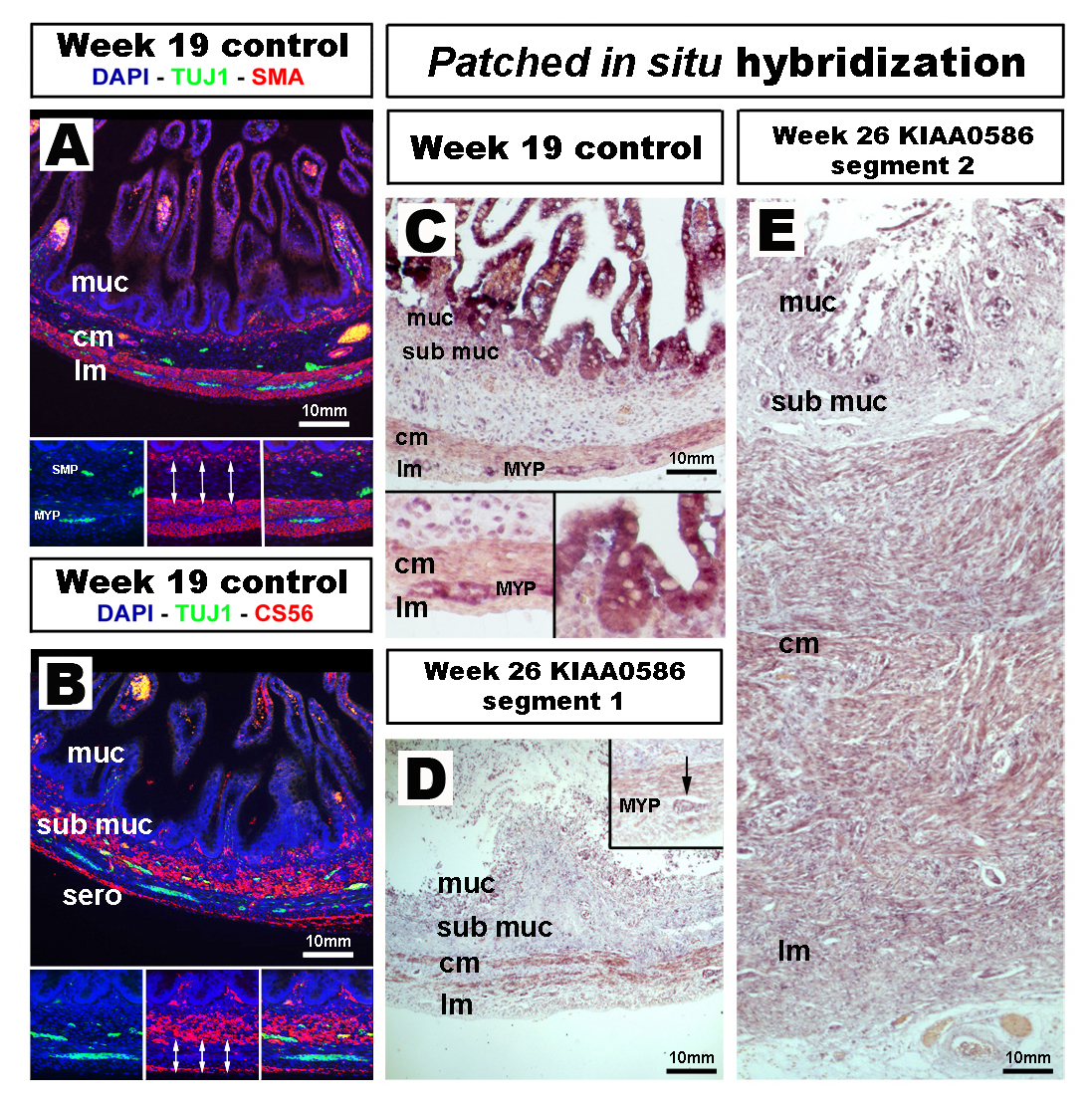

Supplement: Supplementary Figure 5 — Expression of SMA, Tuj-1, CS56 and patched in week 19 control human gut and patched in situ hybridization in 26 weeks KIAA0586 gut. (A,B) Control intestine shows normal neuromuscular patterning and CS56 expression at week 19. (C–E) In situ hybridization against patched in week 19 control and KIAA0586 mutated tissue. (C + insets) Week 19 control intestine shows strong expression of patched in the epithelium, in the myenteric plexus of the ENS and to a lesser extend in the smooth muscle. (D) “Segment 1” shows patched expression in the myenteric plexus (inset) and the muscle layers. (E) “Segment 2” shows a diffuse patched expression throughout the section. muc, mucosa; sub muc, submucosa; cm, circular muscle; lm, longitudinal muscle; MYP, myenteric plexus; sero, serosa. [file Image_5.jpeg]
